# Importance of data structure in comparing two dimension reduction methods for classification of microarray gene expression data

**DOI:** 10.1186/1471-2105-8-90

**Published:** 2007-03-13

**Authors:** Caroline Truntzer, Catherine Mercier, Jacques Estève, Christian Gautier, Pascal Roy

**Affiliations:** 1 CNRS, UMR 5558 – Equipe Biostatistique Santé, Villeurbanne, F-69100, France, Université Claude Bernard Lyon 1, Laboratoire Biostatistique Santé – UMR 5558, Villeurbanne, F-69100, France, Hospices Civils de Lyon, Service de Biostatistique, Lyon, F-69003, France; 2 Université Claude Bernard – Lyon 1, Laboratoire de Biométrie et de Biologie Evolutive – UMR CNRS 5558, Villeurbanne, F-69100, France

## Abstract

**Background:**

With the advance of microarray technology, several methods for gene classification and prognosis have been already designed. However, under various denominations, some of these methods have similar approaches. This study evaluates the influence of gene expression variance structure on the performance of methods that describe the relationship between gene expression levels and a given phenotype through projection of data onto discriminant axes.

**Results:**

We compared Between-Group Analysis and Discriminant Analysis (with prior dimension reduction through Partial Least Squares or Principal Components Analysis). A geometric approach showed that these two methods are strongly related, but differ in the way they handle data structure. Yet, data structure helps understanding the predictive efficiency of these methods. Three main structure situations may be identified. When the clusters of points are clearly split, both methods perform equally well. When the clusters superpose, both methods fail to give interesting predictions. In intermediate situations, the configuration of the clusters of points has to be handled by the projection to improve prediction. For this, we recommend Discriminant Analysis. Besides, an innovative way of simulation generated the three main structures by modelling different partitions of the whole variance into within-group and between-group variances. These simulated datasets were used in complement to some well-known public datasets to investigate the methods behaviour in a large diversity of structure situations. To examine the structure of a dataset before analysis and preselect an a priori appropriate method for its analysis, we proposed a two-graph preliminary visualization tool: plotting patients on the Between-Group Analysis discriminant axis (x-axis) and on the first and the second within-group Principal Components Analysis component (y-axis), respectively.

**Conclusion:**

Discriminant Analysis outperformed Between-Group Analysis because it allows for the dataset structure. An a priori knowledge of that structure may guide the choice of the analysis method. Simulated datasets with known properties are valuable to assess and compare the performance of analysis methods, then implementation on real datasets checks and validates the results. Thus, we warn against the use of unchallenging datasets for method comparison, such as the Golub dataset, because their structure is such that any method would be efficient.

## Background

In cancer research, microarray technology offers a new tool for diagnosis of specific tumors or prognosis of survival. However, in microarray experiments, there are more variables (genes) than samples (patients); if not taken into account, this dimension problem leads to trivial results with no statistical identifiability or biological significance.

Among the methods proposed to overcome this problem, some look for discriminant axes that best separate distinct groups of patients according to specific characteristics. These discriminant axes define a new space whose dimension is lower than that of the original gene space. The discriminant axes are constructed as linear combinations of genes; that is, each gene contributes to the construction of the axes through a coefficient (weight) that depends on its importance in discriminating the groups. Then, for prediction purposes, new patients may be projected in this lower space and assigned to the nearest group. This article focuses on three types of discriminant analysis widely used for prediction purposes: Principal Component Analysis (PCA) followed by Discriminant Analysis (DA), Partial Least Squares followed by DA, and Between-Group Analysis (BGA).

DA is proposed to define discriminant axes [1,2]. One concern in DA is that it is limited by "high dimensionality" and requires a preliminary dimension reduction step. The classical approach to dimension reduction is PCA [3] where components are such that they maximize the gene expression variability across samples. Another approach coming from chemometrics, the PLS method [4-8], selects the components that maximize the covariance between gene expression and phenotype response. To circumvent this preliminary step within the context of microarray data analysis, Culhane *et al*. [9] proposed the Between-Group Analysis [10], because it can be directly used even when the number of variables exceeds the number of samples.

A few recent publications were dedicated to comparisons between projection methods within the context of microarray data analysis. Nguyen and Rocke compared PCA and PLS as prior procedures to logistic discrimination or quadratic discriminant analysis [11]. Boulesteix studied PLS+DA in more detail [12]. Dai *et al*. proposed a new comparison between PCA and PLS extended to a comparison with the Sliced Inverse Regression (SIR) dimension reduction method [13] as prior to logistic discrimination. At the same time, Jeffery *et al*. [14] pointed out that the variance structure of the dataset mostly influences the efficiency and comparison of feature selection methods. No similar work has been done to see whether the structure of the variance of a given dataset may impact the efficiency of the above-cited projection methods. Thus, bioinformaticians may encountered difficulties in choosing the most adapted method for a given dataset.

To solve these difficulties, we found it of major importance to extend the previous comparison studies by a detailed look at the properties of DA -with previous PCA or PLS- and BGA, to understand how some a priori knowledge of the dataset structure may help choosing the most appropriate method.

To achieve this goal, we used both simulated and public well-know datasets in a complementary approach. As to simulated datasets, the article presents a novel simulation process to model various data structures, which leads to different partitions of the whole variance into within-group and between-group variances. A special attention is given to the case where one discriminant axis separates two groups; e.g., whenever a given phenotype classifies the patients into two groups (for example, tumor vs. non-tumor patients). The overall results are discussed to provide appropriate recommendations for more efficient microarray analysis.

## Methods

### General analysis scheme

BGA and DA are based on the same principle: finding one discriminant linear combination of genes that defines a direction in ℝ^*p *^(gene space) along which the between-group variance is maximized. The methodology of multidimensional analysis provides an appropriate framework [15]. Consider a (n * *p*) data array *X *that gives for each *n *patients on rows the values of *p *gene expression levels. Each column, the expression of one gene, is a vector of ℝ^*n *^and each row, the set of gene expression for one patient of the population, is a vector in ℝ^*p*^. The aim was to detect a relationship between patients and genes and find a subspace that provides the best adjustment of the scatter plot. This adjustment requires the definition of a metric in ℝ^*p*^, given by a (*p*, *p*) positive symmetric matrix *Q *that defines a scalar product and distances in ℝ^*p*^.

Introducing information about groups is necessary to find a subspace in which the between-group variance is maximum. This may be reached through introduction of a matrix of indicators *Y*, which enables group identification to be incorporated in a new matrix *Z*. BGA and DA follow the same general analysis scheme using this matrix *Z *and specific choices for *Q *[16].

#### Definition of Z

Let the (*n*, *k*) matrix *Y*, containing *k *class indicators, define a partition of the *n *patients. To maximize the between-group variance, columns of *X *are projected on the subspace defined by the columns of *Y*. This projection is obtained through the projection operator *P*_*Y *_defined as: *P*_*Y *_= *Y*(^*t*^*YY*)^-1^(^*t*^*Y*). Projecting patients on a class of *k *indicators is equivalent to computing the mean expression of each variable in class *k*. *P*_*Y*_*X *is a (*n*, *p*) matrix where the variables for each patient are replaced by the corresponding means of the class he belongs to. Actually, the rank of this matrix is *k *- 1. With this choice of *Z *= *P*_*Y*_*X*, maximizing the variance of a linear combination of *Z *is equivalent to maximizing the between-group variance of *X*. BGA and DA may be seen as a PCA of the mean matrix, each having its own metric in ℝ^*p*^. As said above, BGA does not require a preliminary dimension reduction before projecting patients on the discriminant axis. However, DA requires dimension reduction, which leads first to express patients of *X *in a lower subspace. *X*_*red *_contains the patients coordinates in this reduced space. *Z*_*red *_is then a (*n*, *p*) matrix where variables for each patient in the previously reduced space are replaced by the corresponding means of the class he belongs to.

Two methods are classically proposed to reduce dimension: normed PCA and PLS. They yield components that are linear combinations of genes considered as the new variables to analyze by DA [11]. Each of those components includes all the initial variables weighted according to their contribution to the effect caught by the component. PCA aims at finding components that maximize the projected variance of the data. In contrast, PLS looks directly for components associated with the phenotype. Only a subset of the first components is sufficient to catch most of the data variance or covariance. The optimal number of components was chosen by cross-validation, as described by Boulesteix in the case of PLS+DA [12].

#### Choice of Q

Once *Z *chosen, BGA and DA derive from two distinct choices for *Q*. In BGA, *Q *= *I*_*p *_where *I*_*p *_is the (*p*, *p*) identity matrix. In DA, the metric *Q *= (^*t*^*XX*)^-1^, so the metric involves the total variance-covariance matrix for all patients whatever their group. Another metric could be the mean of the intra-group variances. It corresponds to the so-called Linear Discriminant Analysis. The total variance being the sum of within-group and between-group variances, there is a direct relationship between the two methods. Whatever the metric, the assumption is that variance-covariance matrices are similar in all groups. Moreover, in both cases, the metric involves an inversion of (^*t*^*XX*), which requires not too strongly correlated variables. This is not typically the case in microarray studies due to the huge number of variables, which calls for dimension reduction.

### Statistical solution

The general analysis applies to any pair (*Z*, *Q*). In BGA, the pair is (*Z*, *I*_*p*_) = (*P*_*Y*_*X*, *I*_*p*_); in DA, it is (*Z*_*red*_, (^*t*^*X*_*red*_*X*_*red*_)^-1^). The general scheme aims at finding linear combinations *Zα *maximizing ‖Zα‖In
 MathType@MTEF@5@5@+=feaafiart1ev1aaatCvAUfKttLearuWrP9MDH5MBPbIqV92AaeXatLxBI9gBaebbnrfifHhDYfgasaacH8akY=wiFfYdH8Gipec8Eeeu0xXdbba9frFj0=OqFfea0dXdd9vqai=hGuQ8kuc9pgc9s8qqaq=dirpe0xb9q8qiLsFr0=vr0=vr0dc8meaabaqaciaacaGaaeqabaqabeGadaaakeaadaqbdaqaaiabdQfaAHGaciab=f7aHbGaayzcSlaawQa7amaaBaaaleaacqWGjbqsdaWgaaadbaGaemOBa4gabeaaaSqabaaaaa@359A@, where *α *is a (*p*, *r*) matrix. Those linear combinations define a subspace in which the variance of *Z *is maximum. The single solution is given by singular value decomposition of the matrix *Q*(^*t*^*Z*)*Z*. This matrix can always be diagonalized and has *p *eigenvalues with *r *non-zero ones *λ*_*i*_, *i *= 1...*r*. The *r *corresponding eigenvectors maximize ‖Zα‖In
 MathType@MTEF@5@5@+=feaafiart1ev1aaatCvAUfKttLearuWrP9MDH5MBPbIqV92AaeXatLxBI9gBaebbnrfifHhDYfgasaacH8akY=wiFfYdH8Gipec8Eeeu0xXdbba9frFj0=OqFfea0dXdd9vqai=hGuQ8kuc9pgc9s8qqaq=dirpe0xb9q8qiLsFr0=vr0=vr0dc8meaabaqaciaacaGaaeqabaqabeGadaaakeaadaqbdaqaaiabdQfaAHGaciab=f7aHbGaayzcSlaawQa7amaaBaaaleaacqWGjbqsdaWgaaadbaGaemOBa4gabeaaaSqabaaaaa@359A@ under *Q*^-1^-orthonormality constraint; they are defined in ℝ^*p*^, and called principal factors. Columns of *α *contain these eigenvectors. By definition, the *α*_*i *_are *Q*^-1^-normed. With this construction, linear combinations are uncorrelated.

In the particular case discussed here, where *Z *corresponds to a mean table for two groups, there is only one discriminant axis, so *r *= 1. In the general case of k groups, *r *= *k *- 1.

### Performance estimator

BGA and DA were compared using their predictive performances; i.e., the proportion of correctly classified patients.

The phenotype of a new patient was predicted according to its position on the discriminant axis relative to the threshold defined as:

X¯G1SDG2+X¯G2SDG1SDG1+SDG2     (1)
 MathType@MTEF@5@5@+=feaafiart1ev1aaatCvAUfKttLearuWrP9MDH5MBPbIqV92AaeXatLxBI9gBaebbnrfifHhDYfgasaacH8akY=wiFfYdH8Gipec8Eeeu0xXdbba9frFj0=OqFfea0dXdd9vqai=hGuQ8kuc9pgc9s8qqaq=dirpe0xb9q8qiLsFr0=vr0=vr0dc8meaabaqaciaacaGaaeqabaqabeGadaaakeaadaWcaaqaaiqbdIfayzaaraWaaSbaaSqaaiabdEeahjabigdaXaqabaGccqWGtbWucqWGebardaWgaaWcbaGaem4raCKaeGOmaidabeaakiabgUcaRiqbdIfayzaaraWaaSbaaSqaaiabdEeahjabikdaYaqabaGccqWGtbWucqWGebardaWgaaWcbaGaem4raCKaeGymaedabeaaaOqaaiabdofatjabdseaenaaBaaaleaacqWGhbWrcqaIXaqmaeqaaOGaey4kaSIaem4uamLaemiraq0aaSbaaSqaaiabdEeahjabikdaYaqabaaaaOGaaCzcaiaaxMaadaqadaqaaiabigdaXaGaayjkaiaawMcaaaaa@4B53@

In Equation (1), X¯
 MathType@MTEF@5@5@+=feaafiart1ev1aaatCvAUfKttLearuWrP9MDH5MBPbIqV92AaeXatLxBI9gBaebbnrfifHhDYfgasaacH8akY=wiFfYdH8Gipec8Eeeu0xXdbba9frFj0=OqFfea0dXdd9vqai=hGuQ8kuc9pgc9s8qqaq=dirpe0xb9q8qiLsFr0=vr0=vr0dc8meaabaqaciaacaGaaeqabaqabeGadaaakeaacuWGybawgaqeaaaa@2DFD@_*G*1_, X¯
 MathType@MTEF@5@5@+=feaafiart1ev1aaatCvAUfKttLearuWrP9MDH5MBPbIqV92AaeXatLxBI9gBaebbnrfifHhDYfgasaacH8akY=wiFfYdH8Gipec8Eeeu0xXdbba9frFj0=OqFfea0dXdd9vqai=hGuQ8kuc9pgc9s8qqaq=dirpe0xb9q8qiLsFr0=vr0=vr0dc8meaabaqaciaacaGaaeqabaqabeGadaaakeaacuWGybawgaqeaaaa@2DFD@_*G*2_, *SD*_*G*1 _and *SD*_*G*2 _are respectively the means and standard deviations of the two groups. This threshold was proposed by Culhane *et al*. [9] for BGA and used here also for DA. It allows taking into account the accuracy of the assignment, a greater weight being given to the less scattered group.

Following the idea of Boulesteix [12], Leave-k-Out Cross-Validation was used to obtain the proportion of correctly classified patients. In each loop, the dataset was randomly split so that *k *= 1/3 of the samples were left out and the model derived using the 2/3 samples was applied to predict the class of the remaining samples. This operation was repeated fifty times and a mean misclassification proportion computed. With DA, the selection of the number of components was included in the cross-validation process. The mean misclassification proportion was determined for each number of components used as variables. Finally, the number of components kept was the one for which the misclassification proportion over the fifty runs was minimal.

The variability of the performance estimator (PE) was measured somewhat differently with simulated and real datasets. With simulated datasets and a given set of parameters, the standard deviation of the PE was computed over the fifty simulated datasets. This informs about the variability stemming from the whole process used for PLS+DA, PCA+DA, or BGA. The standard deviation of the PE over the fifty cross-validation runs was computed for each real dataset and for the optimal number of components. This shows to which extent the choice of the split that led to build the training sets may influence the proportion of well-classified samples of the test set, with the same number of components kept.

#### Implementation of methods

All computations were performed using R programming language. The R code that enables to perform simulations is available as additional file [see Additional file 1]. To perform BGA, we used the made4 library [17]. To perform DA with prior PLS or PCA, we relied on the plsgenomics library [18].

### Gene expression datasets

#### DLBCL

This dataset contains 7,129 expression levels on 58 patients with Diffuse Large B-Cell Lymphoma (DLBCL) [19]. After preprocessing and use of a filter method, only 6,149 expression levels were kept. These patients are divided into two subgroups depending on the 5-year survival outcome: 32 "cured" patients and 26 "fatal/refractory" patients. The data are available as .CEL files from the Broad Institute website [20]. The gene expression values were called using the Robust Multichip Average method and data were quantile normalized using the Bioconductor package *affy *[21].

#### Prostate

This dataset provides 102 samples: 50 without and 52 with prostate tumors [22]. The data are available as .CEL files from the Broad Institute website [23]. The gene expression values were obtained as above.

#### ALL

This dataset includes 125 patients with Acute Lymphoblastic Leukemia [24]: 24 patients with and 101 without multidrug resistance (MDR). The pre-processed data are available in the *ALL *library in Bioconductor [21].

#### Leukaemia

This well-known dataset includes expression data on 7,129 genes from 72 tumor-mRNA samples [25]. These acute leukaemia samples belong to two different subtypes of leukaemia: 27 samples categorized as ALL (Acute Lymphoblastic Leukemia) and 45 categorized as AML (Acute Myeloid Leukemia), which is the phenotype of interest. Data are available in the *golubEsets *library in Bioconductor [21]. The data were processed by making the min expression value 100 and the max expression value 16,000. The *log*_2 _of the data was then used.

## Results

The datasets used herein are either artificial data obtained by an original simulation process or the above-cited two-class public datasets.

### Simulated datasets

#### Simulation process

Simulations were performed as a first step to understand the influence of data structure on the results with DA and BGA. An original simulation process was carried out to evaluate the extent to which the above procedures were able to retrieve the structure of a simple two-component problem. We modeled different partitions of the whole variance into within-group and between-group variances using three parameters: i) the variance-covariance structure of each group; ii) the length of the vector joining the barycenters of the two groups; and iii) the direction of this vector, toward a high or a low within-group variance. These three parameters result in several relative positions and eccentricities of the scatter plots in the two-component space.

The simulations started with the generation, in the component space, of two groups with known within-group variances. The maximum dimension of this component space is *n*, the number of patients of the datasets. The between-group difference was expressed in the two-component space. In this space, variables were drawn from a bivariate normal distribution *N*(*μ*, Σ) where Σ is a (2 * 2) diagonal matrix with elements *σ*_1 _and *σ*_2_. *μ *depended on the distance *dist *between the barycenters of the scatter plots.

**Figure 1:**
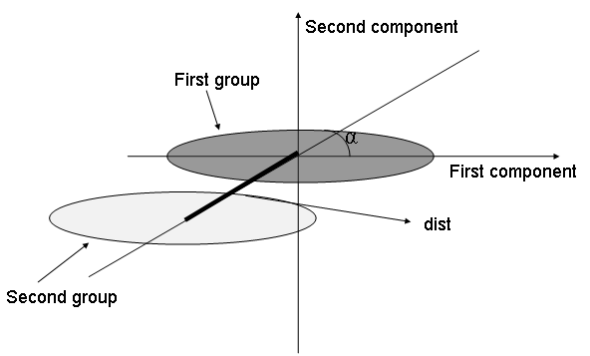
**View of the component space relative to the simulations**. The cluster of points of each of the two groups was plotted in the two-component space. The scatter plots barycenters are distant by *dist*. The direction of the between-group variance draws an angle *α *with the first component.

Thus, *dist *allowed controlling the between-group structure. The chosen ratio *σ*_1_/*σ*_2 _reflects eccentricity: the higher it is, the higher is the eccentricity of the scatterplots; so, this ratio allowed controlling the within-group structure. The line joining the barycenters of the groups and the first component axis forms an angle *α*. Figure 1 shows the geometric meaning of these parameters. The *n *- 2 dimensions left correspond to noise.

Next, patients were expressed in the ℝ^*n *^gene space. For this, gene axes were derived from the component axes through a chosen rotation, which masks more or less the between-group structure present in the two-component space.

The *p *- *n *genes left are random linear combinations of these *n *genes.

#### The effect of dist and α

Table 1 shows prediction results for distances *dist *equal to 1, 3, and 5. The observed differences between DA and BGA did not depend on the previous dimension reduction method, PCA or PLS. However, the number of components kept was always greater (or equal) with PCA than with PLS and, in some cases, this advantaged PCA+DA as seen for *dist *= 1 and *α *= *π*/4, for example.

**Table 1:** Proportion of well-classified patients according to dist, the distance between the barycenters of the two groups

	*α *= *π*/2	*α *= *π*/3	*α *= *π*/4	*α *= *π*/6	*α *= 0
*dist *= 1					
PLS+DA	0.69(0.05)//2	0.69(0.06)//2	0.64(0.06)//2	0.60(0.06)//2	0.59(0.05)//1
PCA+DA	0.69(0.04)//3	0.70(0.05)//2	0.66(0.06)//3	0.60(0.05)//3	0.58(0.06)//1
BGA	0.63(0.05)	0.61(0.06)	0.55(0.06)	0.57(0.05)	0.58(0.05)
*dist *= 3					
PLS+DA	0.93(0.04)//2	0.91(0.03)//2	0.86(0.03)//2	0.71(0.03)//2	0.69(0.06)//1
PCA+DA	0.94(0.04)//2	0.91(0.03)//2	0.85(0.04)//3	0.71(0.04)//3	0.69(0.05)//2
BGA	0.90(0.04)	0.79(0.03)	0.73(0.03)	0.70(0.03)	0.67(0.06)
*dist *= 5					
PLS+DA	0.98(0.04)//2	0.97(0.01)//2	0.97(0.02)//2	0.84(0.03)//2	0.79(0.04)//1
PCA+DA	0.99(0.01)//3	0.98(0.01)//2	0.97(0.02)//2	0.83(0.03)//2	0.79(0.04)//2
BGA	0.91(0.04)	0.91(0.01)	0.86(0.03)	0.82(0.03)	0.79(0.04)

Mean (Standard deviation)//Median of the optimal number of components over fifty datasets simulated with eccentricity such that *ratio *= 10. PLS: Partial Least Squares – PCA: Principal Components Analysis – DA: Discriminant Analysis.

Whatever the method used and the value of *α*, prediction was better as *dist *increased; that is, when the clusters of points were the more distant. Moreover, the more distant the barycenters were, the less the difference between DA and BGA was.

Then, for a given distance, prediction results depended on the value of *α*. The results with DA or BGA were the closest for *α *= *π*/2 and *α *= 0: both inefficient with *α *= 0 and both very efficient with *α *= *π*/2. This corresponded to situations where the between-group direction was simulated on the first or second component axis. For intermediate angles, both methods were less good predictors, with nevertheless an advantage for DA.

#### The effect of eccentricity

Table 2 shows prediction results for several *α *and eccentricities defined by *ratio *= *σ*_1_/*σ*_2_. A *ratio *of 1 corresponds to a spherical cluster of points. As expected, the higher the ratio was, the more advantageous was DA over BGA. Moreover, except for *α *= 0, both methods performed generally better when eccentricity was high. With non-spherical scatter plots, the best prediction was achieved with *α *= *π*/2; that is, when the between-group direction was perpendicular to the within-group direction. When the ratio decreased, DA and BGA got closer, the greatest difference being with *ratio *= 10.

**Table 2:** Proportion of well-classified patients according to ratio, which reflects eccentricity

	*α *= *π*/2	*α *= *π*/3	*α *= *π*/4	*α *= *π*/6	*α *= 0
*ratio *= 10					
PLS+DA	0.82(0.05)//2	0.81(0.03)//2	0.76(0.03)//2	0.71(0.04)//2	0.59(0.04)//2
PCA+DA	0.85(0.05)//3	0.81(0.04)//3	0.77(0.05)//3	0.73(0.05)//3	0.59(0.04)//3
BGA	0.76(0.05)	0.75(0.04)	0.66(0.03)	0.67(0.04)	0.58(0.04)
*ratio *= 2					
PLS+DA	0.68(0.05)//1	0.65(0.04)//1	0.65(0.05)//1	0.65(0.05)//2	0.63(0.05)//1
PCA+DA	0.69(0.05)//3	0.65(0.04)//3	0.67(0.04)//2	0.65(0.04)//2	0.63(0.04)//2
BGA	0.67(0.06)	0.62(0.04)	0.64(0.05)	0.65(.04)	0.62(0.05)
*ratio *= 1					
PLS+DA	0.60(0.05)//2	0.62(0.05)//1	0.64(0.05)//2	0.62(0.05)//1	0.61(0.05)//1
PCA+DA	0.63(0.04)//3	0.63(0.04)//2	0.63(0.05)//2	0.64(0.05)//2	0.63(0.05)//2
BGA	0.61(0.05)	0.62(0.05)	0.61(0.05)	0.61(0.05)	0.60(0.05)

Mean (Standard deviation)//Median of the optimal number of components over over fifty datasets simulated with a distance between the barycenters *dist *= 2. PLS: Partial Least Squares – PCA: Principal Components Analysis – DA: Discriminant Analysis.

Table 3 shows the results when the main components of the group variances were extremely different; that is, when the directions of the principal component of the two clusters of points were perpendicular. In that case, DA and BGA had similar results whatever *α*. Note that PCA was less efficient; in fact, the between-group part was low in the whole variance structure.

**Table 3:** Proportion of well-classified patients with a high eccentricity (ratio = 10) in one group and a low eccentricity (ratio = 0.1) in the other group

	DLBCL	Prostate	ALL	Leukaemia
PLS+DA	0.51(0.14)//12	0.97(0.06)//10	0.73(0.05)//10	0.97(0.03)//1
PCA+DA	0.49(0.09)//13	0.96(0.07)//9	0.57(0.08)//1	0.95(0.04)//5
BGA	0.43(0.10)	0.70(0.09)	0.60(0.06)	0.98(0.03)

Mean (Standard deviation)//Median of the optimal number of components over fifty datasets simulated with *dist *= 2. PLS: Partial Least Squares – PCA: Principal Components Analysis – DA: Discriminant Analysis.

As a general remark, it may be noted that the standard deviation of the performance estimator over the fifty simulated datasets was low whatever the variance partition examined.

Comparable results of simulations were obtained when differences were expressed in two- or three-component spaces.

### Real datasets

The public datasets were chosen to cover the main situations encountered in practice.

To begin the analysis of a new dataset, we suggest to first have a look at its structure to visualize the relative role of the within-group and between-group variances for distinguishing the two groups of patients. For this, we propose two graphs obtained by plotting patients on the BGA discriminant axis (x-axis) and on the first and the second within-group PCA component (y-axis), respectively. The greatest part of the between-group variance is given by the most differential genes, while the other genes tend to mask this between-group structure. For this prior examination of the data structure, we used only the fifty genes with the highest t-test statistics.

**Figure 2:**
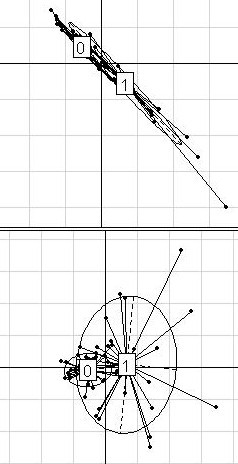
**DLBCL dataset**. Projection of the 58 patients from the DLBCL dataset (32 "cured" and 26 "fatal/refractory") on the discriminant axis obtained with BGA (x-axis), along their coordinates on the first (on the top) and the second (on the bottom) within-group PCA component (y-axis), respectively. For a better legibility, the groups were labeled 0 (for "cured" patients) and 1 (for "fatal/refractory" patients). Only the 50 most differential genes among 6149 were used for these graphs.

**Figure 3:**
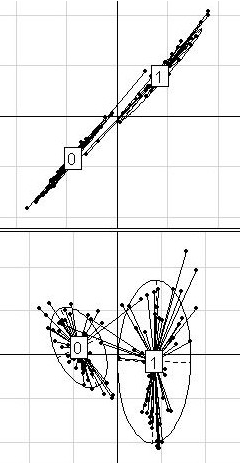
**Prostate dataset**. Projection of the 102 patients from the prostate dataset (50 without and 52 with tumor) on the discriminant axis obtained with BGA (x-axis), along their coordinates on the first (on the top) and the second (on the bottom) within-group PCA component (y-axis), respectively. For a better legibility, the groups were labeled 0 (for non-tumor prostate samples) and 1 (for tumor prostate samples). Only the 50 most differential genes among 12625 were used for these graphs.

**Figure 4:**
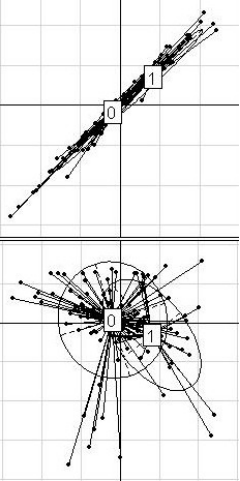
**ALL dataset**. Projection of the 125 patients from the ALL dataset (24 with and 101 without Multi Drug Resistance -MDR-) on the discriminant axis obtained with BGA (x-axis), along their coordinates on the first (on the top) and the second (on the bottom) within-group PCA component (y-axis), respectively. For a better legibility, the groups were labeled 0 (for patients with MDR) and 1 (for patients without MDR). Only the 50 most differential genes among 12625 were used for these graphs.

**Figure 5:**
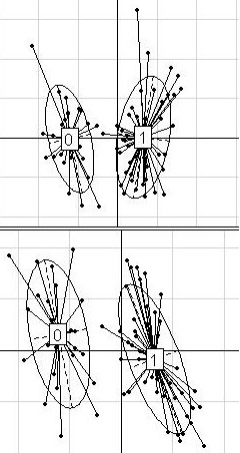
**Leukemia dataset**. Projection of the 72 patients from the leukaemia dataset (25 Acute Lymphoblastic Leukemia -ALL- and 47 Acute Myeloide Leukemia -AML-) on the discriminant axis obtained with BGA (x-axis), along their coordinates on the first (on the top) and the second (on the bottom) within-group PCA component (y-axis), respectively. For a better legibility, the groups were labeled 0 (for AML patients) and 1 (for ALL patients). Only the 50 most differential genes among 7129 were used for these graphs.

Figures 2 to 5 show the plots that correspond to each dataset. In the case of the DLBCL dataset (Figure 2), the clusters of points were not discrete; the cluster relative to the cured patients was even found within the "fatal/refractory" cluster. This suggests that the dataset has no obvious between-group structure. Moreover, the main components of the variances in each group were very different.

In the case of the prostate dataset (Figure 3), the distinction between non-tumor and tumor samples was found along both between-groups and the first within-group directions.

In the case of the ALL dataset (Figure 4), the distinction between patients with or without multidrug resistance (MDR) was found along the first within-group direction.

At last, in the case of the leukaemia dataset (Figure 5), the barycenters were only separated by the between-group direction. This indicates that the between-group direction was perpendicular to the within-group direction.

So, these four datasets reflect various structures of variance; these structures may be associated to simulated datasets to see how their main characteristics explain the predictive behaviour of the methods. Table 4 shows the proportion of well-classified patients obtained over the fifty cross-validation runs with the optimal number of components. The standard deviation of the performance estimator over the fifty cross-validation runs was low. This standard deviation shows the variability of the performance estimator between cross-validation runs. Here, it indicated that the way of splitting patients into training and test sets within each run did not affect the results.

**Table 4:** Proportion of well-classified patients with real datasets

	PLS+DA	PCA+DA	BGA
DLBCL	0.51(0.14)//12	0.49(0.09)//13	0.43(0.10)
Prostate	0.97(0.06)//10	0.96(0.07)//9	0.70(0.09)
ALL	0.73(0.05)//10	0.57(0.08)//1	0.60(0.06)
Leukaemia	0.97(0.03)//1	0.95(0.04)//5	0.98(0.03)

Mean (Standard deviation) over the fifty cross-validation runs for the optimal number of component (indicated after //). Results were obtained with the DLBCL, the prostate, the ALL, and the leukaemia datasets. PLS: Partial Least Squares – PCA: Principal Components Analysis – DA: Discriminant Analysis.

As expected in the light of the structure visualization, the proportions of well-classified samples for the DLBCL were low whatever the method used, BGA being the less efficient. In fact, DA needed 12 PLS components or 13 PCA components to optimize prediction while, with only one component, BGA is not able to catch more information given by the within-group structure. This corresponded in the simulated datasets to a low value of *dist*.

As to the prostate dataset, the plots led to compare this dataset to the case where *α *is intermediate between 0 and *π*/2. Thus, we could foretell that the results would be improved in comparison with those of the DLBCL dataset, and that DA will be more advantageous. Indeed, this was confirmed with the proportions of well-classified samples: DA was more efficient in predicting non-tumor or tumor samples. It seemed that the high number of components kept for the first dimension reduction allowed getting more information than a single projection in BGA.

The ALL dataset corresponded to simulating *α *near to 0; none of the methods was really adapted to such a configuration. Actually, no methods was sufficiently efficient. PCA as first dimension reduction method was not able to catch information. On the contrary, with 10 PLS components, DA overcame BGA.

As to the leukaemia dataset, it recalled the simulated case with *α *= *π*/2, which is the one that allowed the best results. This was confirmed in Table 4, where the three methods were particularly efficient in distinguishing ALL and AML patients. The prediction results obtained with BGA and DA were very similar. With dimension reduction, one PLS component and five PCA components were needed to optimize prediction. The results with PCA suggested that the between-group variance took the largest part of the total variance.

Further figures are provided as additional files showing the structure of other well-known datasets: DLBCL vs FL [see Additional file 2], Colon (normal vs tumor samples) [see Additional file 3], Myeloma (With vs without lytic lesions) [see Additional file 4], ALL1 (B-Cell vs T-Cell origin) [see Additional file 5], ALL2 (Relapse vs no relapse) [see Additional file 6], ALL3 (With vs without t(9;22) translocation) [see Additional file 7]. The corresponding proportions of well-classified patients obtained over the fifty cross-validation runs with the optimal number of components are provided in additional file 8 [see Additional file 8].

## Discussion

Results from both simulated and real datasets showed that the structure of a dataset influences to a large extent the efficiency of the methods that use projection on discriminant axes.

In testing a new method, simulated and real datasets play complementary roles. Simulation of data with known properties is useful to study the influence of the dataset characteristics and the performance of a given method, and could be considered as a practical guide to understand results from real situations. For choosing an analysis method to discriminate two groups of patients, we think it is necessary to have a prior examination of the structure of the data to analyze. This will enable an informed choice between the available methods.

We propose here a new simulation approach that allows exploring known structures with control through several parameters. Nguyen [26] proposed to simulate datasets to compare the performance of PCA and PLS as prior procedure before logistic discrimination. However, his method of simulation did not allow a discussion on the influence of the data structure. Our simulations allow generating different structures of different degrees of complexity and assessing the impact of three parameters: the distance between the clusters, the eccentricity of these clusters, and their relative positions in a two-dimensional component space. The major source of complexity in real microarray datasets is the existence of regulation networks. In our simulations, this may be described by a component with a very large variance; that is, a large eccentricity. This corresponds usually to a common effect on all the genes. A high variance on one component corresponds also to a cluster of highly correlated genes. Whether a network of genes exists or not would determine the relative importance of the other components with respect to the first one. Nevertheless, we are aware that our simulations have limits. Therefore, a compromise has to be found between the uncontrolled nature of real datasets and the controlled nature of simulated datasets as research tools. This will be the object of future works.

The use of real datasets to prove the superiority of any method should be considered with caution. For example, the leukaemia dataset from Golub, very often used to demonstrate the efficiency of a new method, may not be used for that purpose because of its very strong between-group structure. This structure is such that we expect the groups to be distinguished whatever the method used (e.g., BGA that simply joins the barycenters of the groups). We believe that, in such situations, the good performance of a particular method does not only inform on its ability to discriminate between groups. If the structure of the dataset had been previously examined before its analysis, for example with the graphical tool we propose, this dataset would not have been chosen to validate new prediction methods. Thus, bioinformaticians should be cautious in choosing the datasets to use for method comparisons. The proposed visualization tool helps in choosing the dataset, by having an idea of its structure. The prostate or ALL datasets for example may be appropriate for that purpose.

Besides, the structure of a given dataset may depend on the type of disease. In diagnosis, some pathophysiological entities may be already clearly identified; if their origin is a metabolic activation, they will induce different processes that will be easy to distinguish (e.g., ALL vs. AML). However, differentiating patients with or without multidrug resistance may be even more difficult because no pathophysiological entities are involved. In prognosis, distinguishing good from bad prognosis patients would be more difficult because they often share the same pathophysiological characteristics.

Three main configurations of the data structure may be identified. When the clusters of points are quite distinct the between-group difference is so obvious that the within-group structure will have no impact; BGA and DA will give good prediction results. The simple method that consists in drawing an axis between the barycenters is sufficient. In fact, the way of projecting patients on the discriminant axis does not come into consideration. On the opposite, there are situations in which both methods are inappropriate. This corresponds to superposed clusters of points obtained in plotting the within-group versus the between-group coordinates. In other situations, we believe that DA is more advantageous than BGA because it allows taking into account the partition of the total variance into between and within variances. However, in case the variances of the two groups are not the same, the total variance will not reflect the variance in each group, so there will be no advantage of favoring DA over BGA. Moreover, keeping more than one component in the first dimension reduction step using PLS or PCA is a way to capture more information than the single projection in BGA, particularly with PLS. This is illustrated with the ALL dataset; by keeping ten PLS components, DA outperforms BGA to a large extent (respectively 0.97% and 0.70% of well-classified patients). These observations illustrate the fact that the first PLS component and the BGA discriminant axis are identical. This was demonstrated by Barker and Rayens [27], and by Boulesteix [12]. Thus, using PLS with one component followed by DA gives a final component that is collinear to that of PLS alone, and also to the BGA axis. This is illustrated with the leukaemia dataset, where PLS+DA and BGA give equivalent results (respectively 0.97% and 0.98% of well-classified patients). However, in simulations, PLS+DA seemed to yield, on average, slightly better results than BGA. In fact, due to random sampling, some simulated datasets needed more than one component to optimize prediction because dimensions other than those simulated may be informative by chance alone. Note that in case of a spherical cluster of points, a second PLS component will not capture more information than the first one and both methods will be equally efficient.

Overall, DA becomes advantageous when the structure of the variance is such that the way of projecting patients on the discriminant axis needs to come into consideration. This leads to conclude that DA is the most suitable method; it provides better or at least equivalent results in a diversity of datasets because it ensures that the within-group variance will be taken into account, when relevant. The diversity of real datasets encountered confirms the fact that, unlike DA, BGA is unable to deal with too complex data structures. The only advantage of BGA is its ease of use and interpretation: a single projection enables to go from the original variable space to a one-dimension axis on which inter-group variance is maximum.

This axis is also a direct linear combination of genes where a high coefficient means that the gene is important to classify the patients into one of the groups. With DA, the samples are first expressed in a component space, which makes interpretation more difficult.

BGA and DA used with more than two groups provide *k *- 1 discriminant axes, which enables each of the *k *groups to be separated from the *k *- 1 others. By plotting these groups in successive two-dimensional graphs, the structure assessment described here may be applied to each of the two-dimension spaces so obtained.

## Conclusion

We have established here that the two methods -BGA and DA with prior PCA or PLS- are based on very similar approaches. Efficient use of these projection methods requires some a priori knowledge of the structure of the clusters of points. We found that three main structure situations may be identified. When the clusters of points are clearly split, both methods will perform equally well and it becomes futile to prove the superiority of one method over the other using datasets previously shown of simple structure. When the clusters of points superpose, both methods will fail to yield interesting predictions. In such a case, there is no linear way to separate groups, leading to the use of non linear methods. In intermediate situations, the structure of the clusters of points has to be taken into account by the projection to improve prediction, which imposes the use of DA. So, we recommend the use of Discriminant Analysis to take into account more diverse dataset structures.

## Authors' contributions

CT wrote the computer code for simulations, carried out the analysis, analyzed the results and drafted the manuscript. JE and PR contributed to simulations design, result interpretation, and contributed with CM and GC to write the manuscript. All authors read and approved the final manuscript.

## Supplementary Material

Additional file 1 **R codes used to generate simulated datasets**. This simulation process generates several datasets structures by modelling different partitions of the whole variance into within-group and between-group variances. Click here for file

Additional file 2 **DLBCL vs FL dataset**. Projection of 58 patients with Diffuse Large B-Cell Lymphoma and 19 patients with Follicular Lymphoma on the discriminant axis obtained with BGA (x-axis), along their coordinates on the first (on the top) and the second (on the bottom) within-group PCA component (y-axis), respectively. For a better legibility, the groups were labeled 0 (for FL-patients) and 1 (for DLBCL-patients). Only the 50 most differential genes among 7129 were used for these graphs. The data are available from the Broad Institute website [20]. Click here for file

Additional file 3 **Colon dataset**. Projection of 22 normal controls and 40 tumor samples on the discriminant axis obtained with BGA (x-axis), along their coordinates on the first (on the top) and the second (on the bottom) within-group PCA component (y-axis), respectively. For a better legibility, the groups were labeled 0 (normal controls) and 1 (for tumor samples). Only the 50 most differential genes among 2000 were used for these graphs. The data are available in the *ColonCA *library in Bioconductor [21]. Click here for file

Additional file 4 **Myeloma dataset**. Projection of 36 patients with and 137 patients without lytic lesions on the discriminant axis obtained with BGA (x-axis), along their coordinates on the first (on the top) and the second (on the bottom) within-group PCA component (y-axis), respectively. For a better legibility, the groups were labeled 0 (lytic lesions) and 1 (without lytic lesions). Only the 50 most differential genes among 12625 were used for these graphs. Data can be download from Gene Expression Omnibus [28] (accession number GDS531). Click here for file

Additional file 5 **ALL1 dataset**. Projection of 95 Acute Lymphoblastic Leukaemia (ALL) patients with B-Cell and 33 with T-Cell origin on the discriminant axis obtained with BGA (x-axis), along their coordinates on the first (on the top) and the second (on the bottom) within-group PCA component (y-axis), respectively. For a better legibility, the groups were labeled 0 (B-Cell) and 1 (T-Cell). Only the 50 most differential genes among 12625 were used for these graphs. The data are available in the *GOstats *library in Bioconductor [21]. Click here for file

Additional file 6 **ALL2 dataset**. Projection of 65 ALL patients that did and 35 that did not relapse on the discriminant axis obtained with BGA (x-axis), along their coordinates on the first (on the top) and the second (on the bottom) within-group PCA component (y-axis), respectively. For a better legibility, the groups were labeled 0 (no relapse) and 1 (relapse). Only the 50 most differential genes among 12625 were used for these graphs. The data are available in the *GOstats *library in Bioconductor [21]. Click here for file

Additional file 7 **ALL3 dataset**. Projection of 26 ALL-patients with and 67 ALL-patients without the t(9;22) translocation on the discriminant axis obtained with BGA (x-axis), along their coordinates on the first (on the top) and the second (on the bottom) within-group PCA component (y-axis), respectively. For a better legibility, the groups were labeled 0 (without t(9;22)) and 1 (with t(9;22)). Only the 50 most differential genes among 12625 were used for these graphs. The data are available in the *GOstats *library in Bioconductor [21]. Click here for file

Additional file 8 **Proportion of well-classified patients for complementary two-class real datasets**. Mean (Standard Deviation) over the fifty cross-validation runs for the optimal number of component (indicated after //). The table shows results for the following datasets: DLBCL vs FL, Colon, Myeloma, ALL1, ALL2, and ALL3. Click here for file
