# Supplementary material for: Importance of data structure in comparing two dimension reduction methods for classification of microarray gene expression data
Source: BMC Bioinformatics. 2007 Mar 13;8:90. doi: 10.1186/1471-2105-8-90 (PMC1831790; doi:10.1186/1471-2105-8-90)
Supplement: Additional file 1 — R codes used to generate simulated datasets. This simulation process generates several datasets structures by modelling different partitions of the whole variance into within-group and between-group variances. [file 1471-2105-8-90-S1.pdf]

```
#######################################
#   R functions to generate simulated datasets.
#######################################

library(MASS)

##nbsimul is the number of datasets to be simulated.
##dist is the distance between barycenters
##var 1 to var 4 are the components of Sigma

simulations=function(nbsimul,dist, var1, var2,var3,var4, alpha, angle1, angle2)
{
CreateData=function(dist, var1, var2,var3, var4, alpha, angle1, angle2)
{
##Generation of two groups in the component space
cor1=matrix(c(var1, 0,0,var2), ncol=2)
cor2=matrix(c(var2, 0,0,var3), ncol=2)
gpe1=mvrnorm(50, c(0, 0), cor1)
abc=dist*cos(alpha)
ord=dist*sin(alpha)
gpe2=mvrnorm(50, c(-abc,-ord), cor2)
Comp2=rbind(gpe1,gpe2)

##Generation of the n-2 left components that correspond to noise
AutresComp=matrix(rep(0,48*100), nc=48)
cor=matrix(rep(0,48*48),nr=48)
diag(cor)=1
AutresComp=mvrnorm(100, rep(0,48),cor)
TousComp=cbind(Comp2,AutresComp

##Expression of individuals in the R^n space
A=matrix(runif(50*50,min=-1.5, max=1.5), ncol=50)
A[1:2,1]=c(sin(angle1),cos(angle1))
A[-(1:2),1]=rep(0, length(A[-(1:2),1]))
A[1:3,2]=c(-cos(angle1)*cos(angle2), sin(angle1)*cos(angle2),sin(angle2))
A[-(1:3),2]=rep(0, length(A[-(1:3),2]))

B=schmidt(A)
f2 <- function(v) sqrt(sum(v * v ))
B.var <- apply(B, 2, f2)
Bb <- sweep(B, 2,B.var, "/")
Coord.Genes=TousComp%*%t(Bb)

##Generation of the p-n gene expression levels, random combinations of the n first genes.
coeff.mat=matrix(runif(450*50, min=-1,max=1),ncol=450)
AutresGenes=Coord.Genes%*%coeff.mat
TousGenes=cbind(Coord.Genes,AutresGenes)
}
res=list(NA,nbsimul)
res=replicate(nbsimul,CreateData(dist, var1, var2,var3, var4, alpha, angle1, angle2), simp=F)
res
}

schmidt=function(A)
{
n<-dim(A)
for (i in 2:n[2])
        {
        for (j in 1:(i-1))
                {
                A[,i] <- A[,i] - sum(A[,i]*A[,j])/sum(A[,j]*A[,j])*A[,j]
                }
        }
return(A)
}


#######################################
#   Example for running “simulations”
#   function
#######################################

dataList =simulations (50,5,10,1,10,1,pi/2, pi/4, pi/3)
##dataList is a list of 50 simulated datasets.
```
