# Supplementary figures and images for: Importance of data structure in comparing two dimension reduction methods for classification of microarray gene expression data
Source: BMC Bioinformatics. 2007 Mar 13;8:90. doi: 10.1186/1471-2105-8-90 (PMC1831790; doi:10.1186/1471-2105-8-90)

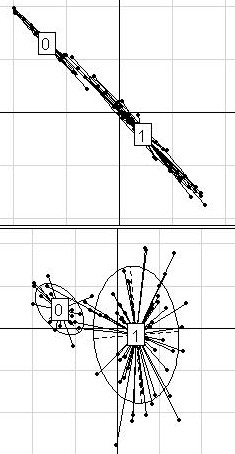

Supplement: Additional file 2 — DLBCL vs FL dataset. Projection of 58 patients with Diffuse Large B-Cell Lymphoma and 19 patients with Follicular Lymphoma on the discriminant axis obtained with BGA (x-axis), along their coordinates on the first (on the top) and the second (on the bottom) within-group PCA component (y-axis), respectively. For a better legibility, the groups were labeled 0 (for FL-patients) and 1 (for DLBCL-patients). Only the 50 most differential genes among 7129 were used for these graphs. The data are available from the Broad Institute website [20]. [file 1471-2105-8-90-S2.jpeg]

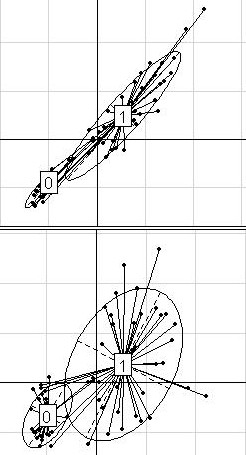

Supplement: Additional file 3 — Colon dataset. Projection of 22 normal controls and 40 tumor samples on the discriminant axis obtained with BGA (x-axis), along their coordinates on the first (on the top) and the second (on the bottom) within-group PCA component (y-axis), respectively. For a better legibility, the groups were labeled 0 (normal controls) and 1 (for tumor samples). Only the 50 most differential genes among 2000 were used for these graphs. The data are available in the ColonCA library in Bioconductor [21]. [file 1471-2105-8-90-S3.jpeg]

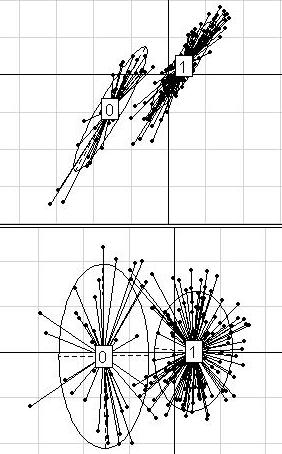

Supplement: Additional file 4 — Myeloma dataset. Projection of 36 patients with and 137 patients without lytic lesions on the discriminant axis obtained with BGA (x-axis), along their coordinates on the first (on the top) and the second (on the bottom) within-group PCA component (y-axis), respectively. For a better legibility, the groups were labeled 0 (lytic lesions) and 1 (without lytic lesions). Only the 50 most differential genes among 12625 were used for these graphs. Data can be download from Gene Expression Omnibus [28] (accession number GDS531). [file 1471-2105-8-90-S4.jpeg]

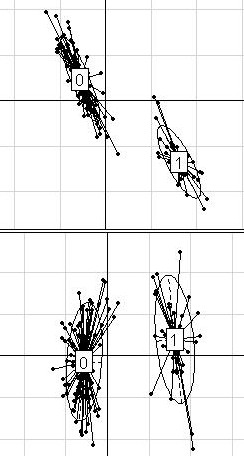

Supplement: Additional file 5 — ALL1 dataset. Projection of 95 Acute Lymphoblastic Leukaemia (ALL) patients with B-Cell and 33 with T-Cell origin on the discriminant axis obtained with BGA (x-axis), along their coordinates on the first (on the top) and the second (on the bottom) within-group PCA component (y-axis), respectively. For a better legibility, the groups were labeled 0 (B-Cell) and 1 (T-Cell). Only the 50 most differential genes among 12625 were used for these graphs. The data are available in the GOstats library in Bioconductor [21]. [file 1471-2105-8-90-S5.jpeg]

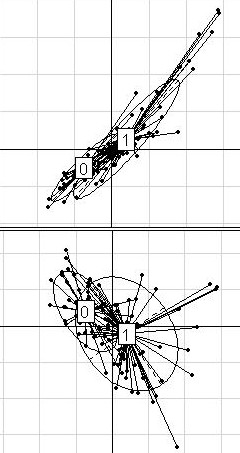

Supplement: Additional file 6 — ALL2 dataset. Projection of 65 ALL patients that did and 35 that did not relapse on the discriminant axis obtained with BGA (x-axis), along their coordinates on the first (on the top) and the second (on the bottom) within-group PCA component (y-axis), respectively. For a better legibility, the groups were labeled 0 (no relapse) and 1 (relapse). Only the 50 most differential genes among 12625 were used for these graphs. The data are available in the GOstats library in Bioconductor [21]. [file 1471-2105-8-90-S6.jpeg]

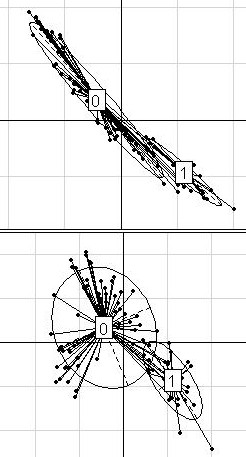

Supplement: Additional file 7 — ALL3 dataset. Projection of 26 ALL-patients with and 67 ALL-patients without the t(9;22) translocation on the discriminant axis obtained with BGA (x-axis), along their coordinates on the first (on the top) and the second (on the bottom) within-group PCA component (y-axis), respectively. For a better legibility, the groups were labeled 0 (without t(9;22)) and 1 (with t(9;22)). Only the 50 most differential genes among 12625 were used for these graphs. The data are available in the GOstats library in Bioconductor [21]. [file 1471-2105-8-90-S7.jpeg]
