# Supplementary material for: Importance of data structure in comparing two dimension reduction methods for classification of microarray gene expression data
Source: BMC Bioinformatics. 2007 Mar 13;8:90. doi: 10.1186/1471-2105-8-90 (PMC1831790; doi:10.1186/1471-2105-8-90)
Supplement: Additional file 8 — Proportion of well-classified patients for complementary two-class real datasets. Mean (Standard Deviation) over the fifty cross-validation runs for the optimal number of component (indicated after //). The table shows results for the following datasets: DLBCL vs FL, Colon, Myeloma, ALL1, ALL2, and ALL3. [file 1471-2105-8-90-S8.pdf]

	PLS+DA	PCA+DA	BGA
Colon	0.87(0.06) //2	0.83(0.06) //5	0.88(0.06)
DLBCL vs FL	0.97(0.03) //3	0.96(0.03) //10	0.84(0.08)
Myeloma	0.79(0.10) //1	0.72(0.05) //12	0.78(0.04)
ALL1	0.99(0.01) //2	0.99(0.01) //5	0.99(0.01)
ALL2	0.57(0.07) //6	0.59(0.08) //1	0.52(0.07)
ALL3	0.82(0.07) //4	0.59(0.08) //6	0.73(0.09)
